# The Postural Tachycardia Syndrome (POTS): Pathophysiology, Diagnosis & Management

**Published:** 2006-04-01

**Authors:** Satish R Raj

**Affiliations:** Autonomic Dysfunction Center, Division of Clinical Pharmacology, Departments of Medicine & Pharmacology, Vanderbilt University, Nashville, Tennessee, USA

**Keywords:** Postural Tachycardia Syndrome, Pathophysiology, Diagnosis, Management

## Abstract

Postural tachycardia syndrome (POTS), characterized by orthostatic tachycardia in the absence of orthostatic hypotension, has been the focus of increasing clinical interest over the last 15 years 1. Patients with POTS complain of symptoms of tachycardia, exercise intolerance, lightheadedness, extreme fatigue, headache and mental clouding. Patients with POTS demonstrate a heart rate increase of ≥30 bpm with prolonged standing (5-30 minutes), often have high levels of upright plasma norepinephrine (reflecting sympathetic nervous system activation), and many patients have a low blood volume. POTS can be associated with a high degree of functional disability. Therapies aimed at correcting the hypovolemia and the autonomic imbalance may help relieve the severity of the symptoms. This review outlines the present understanding of the pathophysiology, diagnosis, and management of POTS.

## Introduction

Postural tachycardia syndrome (POTS), characterized by orthostatic tachycardia in the absence of orthostatic hypotension, has been the focus of increasing clinical interest over the last 15 years [1]. Patients with POTS complain of symptoms of tachycardia, exercise intolerance, lightheadedness, extreme fatigue, headache and mental clouding. This disorder is not new [2], but has gone by many different names over the last 150 years, including mitral valve prolapse syndrome, neurocirculatory asthenia, orthostatic tachycardia, and orthostatic intolerance [3,4]. An advantage of the name postural tachycardia syndrome (POTS) is that it focuses attention on the sympathetic activation which characterizes the disorder. This review outlines the present understanding of the pathophysiology, diagnosis, and management of POTS.

## Physiology of Upright Posture

Assumption of the upright posture requires prompt physiological adaptation to gravity. There is an instantaneous descent of ~500 ml of blood from the thorax to the lower abdomen, buttocks, and legs. In addition, there is a 10-25% shift of plasma volume out of the vasculature and into the interstitial tissue [5]. This shift decreases venous return to the heart, resulting in a transient decline in both arterial pressure and cardiac filling. This has the effect of reducing the pressure on the baroreceptors, triggering a compensatory sympathetic activation that results in an increase in heart rate and systemic vasoconstriction (countering the initial decline in blood pressure). Hence, assumption of upright posture results in a 10-20 beat per minute increase in heart rate, a negligible change in systolic blood pressure, and a ~5 mmHg increase in diastolic blood pressure.

## Pathophysiology of Orthostatic Dysregulation

Failure of the regulatory mechanism to respond properly may lead to either ***orthostatic hypotension***, as is seen in autonomic failure, or ***orthostatic tachycardia***, as is seen in POTS. Orthostatic hypotension is defined as a fall in pressure on standing of more than 20/10 mmHg. However, it is common in patients with autonomic failure for the decline to be much greater than this, which may result in loss of consciousness soon after standing. On the other hand, in POTS, blood pressure is typically maintained on standing or may even increase. Heart rate rises more than 30 bpm and symptoms reminiscent of impaired cerebral perfusion may develop.

## Clinical Presentation of Postural Tachycardia Syndrome (POTS)

### Diagnostic Criteria & Common Clinical Features

POTS is defined (Table 1) as the presence of symptoms of orthostatic intolerance for at least 6 months accompanied by a heart rate increase of at least 30 beats/min within 5-30 minutes of assuming an upright posture. This should occur in the absence of orthostatic hypotension (a fall in blood pressure >20/10 mmHg). The syndrome must occur in the absence of prolonged bed rest, medications that impair autonomic regulation (such as vasodilators, diuretics, antidepressants or anxiolytic agents), or any other chronic debilitating disorders that might cause tachycardia (such as dehydration, anemia or hyperthyroidism). It is important to recognize that this syndrome is typically disabling. Hence, the mere observation of orthostatic tachycardia is not, by itself, sufficient to make the diagnosis of POTS.

Symptoms include mental clouding (“brain fog”), blurred or tunneled vision, shortness of breath, palpitation, tremulousness, chest discomfort, headache, lightheadedness and nausea. While pre-syncope is common in these patients, only a minority (~30%) actually pass out. The chest pains are almost never due to coronary artery obstruction, but are sometimes associated with electrocardiographic changes in the inferior leads, particularly when upright [6].

Many patients complain of significant exercise intolerance and extreme fatigue. Even activities of daily living, such as bathing or housework, may greatly exacerbate symptoms with resultant fatigue. This can pose significant limitations on their functional capacity.

The disorder primarily affects women of child-bearing age. The female:male ratio is 4:1. The reason for the strong female predominance is not known, but it should be noted that orthostatic tolerance is reduced in normal healthy females [7]. Others disorders such as autoimmune diseases and irritable bowel syndrome are seen commonly in patients with POTS, and also have higher prevalence in women.

Patients frequently report that their symptoms began following acute stressors such as pregnancy, major surgery, or a presumed viral illness, but in others cases, symptoms develop more insidiously. About 80% of female patients report an exacerbation of symptoms in the pre-menstrual phase of their ovulatory cycle (unpublished data). Gazit et al. have also reported an association between joint hypermobility and POTS [8]. Many patients have bowel irregularities and have been co-diagnosed with irritable bowel syndrome, and some have abnormalities of sudomotor regulation [9].

### Psychological Profile in POTS

Patients with POTS are sometimes clinically diagnosed as having anxiety disorders such as panic disorder. Indeed, patients demonstrate elevated scores on the Beck Anxiety Inventory [10] (23±10 vs. 7±8; P<0.001), a commonly used instrument that quantifies the magnitude of anxiety symptoms [11]. Unfortunately, this questionnaire includes somatic anxiety symptoms (such as palpitation) which can result from a hyperadrenergic state such as is seen in POTS. When a newer, cognitive-based measure of anxiety (the Anxiety Sensitivity Index [12]) is used, there was a trend toward less anxiety in the patients with POTS than the general population (15±10 vs. 19±9; P=0.063) [11]. Thus, much of the anxiety attributed to patients with POTS might be due to a misinterpretation of their physical symptoms.

We did find that patients with POTS often have diminished attention and concentration compared to matched healthy volunteers [11]. Using the Inattention score from the Connors Adult ADHD Rating Scale [13], the patients with POTS scored significantly higher than did the normal control subjects.

### Physical Findings in POTS

**Figure 1:**
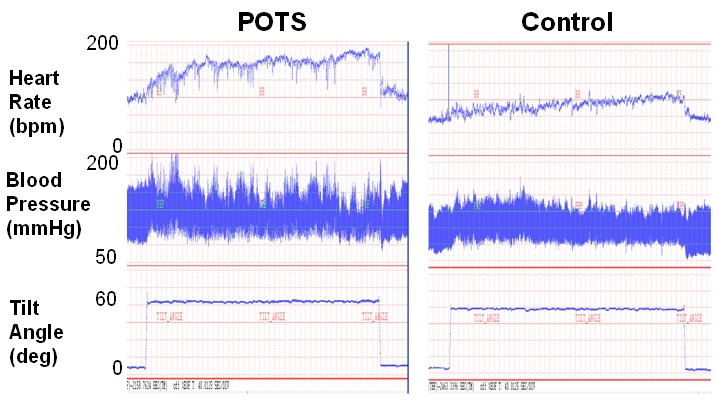
Hemodynamics with Upright Posture in POTS The tracings for heart rate, blood pressure, and tilt table angle are shown for a patient with the postural tachycardia syndrome (POTS; **left**) and for a healthy control subject (**right**) during a 30 minute tilt head-up test. With head-up tilt, the heart rte immediately increases in POTS and peaks at over 170 bpm prior to the end of the tilt. In contrast the heart rate of the healthy control subject rises to just over 100 bpm. The patient with POTS does not experience a reduction in blood pressure during the tilt test. It is largely unchanged during the test.

The most striking physical feature of POTS is the severe tachycardia that develops on standing from a supine position. Blood pressure and heart rate must be measured in both postures and should be taken not only immediately after standing but also at 2, 5 and 10 minutes as occasional patients have a delayed tachycardia [14]. Normal subjects commonly develop a transient tachycardia within the 1st minute of standing that should not be mistaken for POTS. A sustained heart rate increase ≥30 beats per minute is considered diagnostic of orthostatic tachycardia (Figure 1). The systolic blood pressure should not fall by more than 20 mmHg, and in many cases it will actually increase with standing. Recent data suggests that there may be a significant circadian variability in the orthostatic tachycardia seen in patients with POTS [15]. In a cohort of 17 patients with POTS, the orthostatic tachycardia was greater in the morning than in the evening (38±4 bpm vs. 27±3 bpm; P<0.001), while there was no diurnal difference in the orthostatic change in blood pressure. These data suggest that to optimize diagnostic sensitivity, postural vital signs should be performed in the morning.

**Figure 2:**
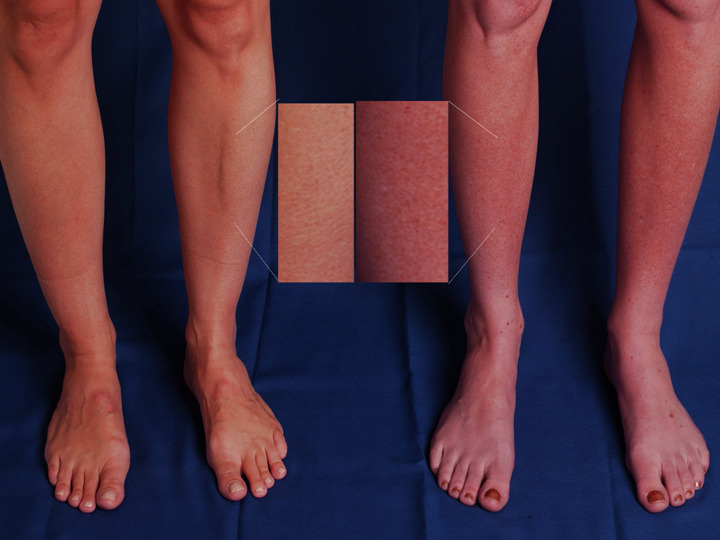
Acrocyanosis in POTS One of the more striking physical features in the postural tachycardia syndrome (POTS) is the gross change in dependent skin color that can occur with standing. The panel shows the legs of 2 people who have been standing for 5 minutes, a healthy control subject (**left**) and a patient with POTS (**right**). The patient with POTS (right) has significant dark red mottling of her legs extending up to the knees while standing, while the control subject does not have a similar discoloration.

Cardiac auscultation may reveal a murmur of mitral valve prolapse, but significant mitral regurgitation is unusual. A striking physical feature of POTS is the dependant acrocyanosis that occurs in 40-50% of patients with POTS (Figure 2). These patients experience a dark red-blue discoloration of their legs, which are cold to the touch. This can extend from the feet to above the level of the knees. The reasons underlying this phenomenon are not clear. The current data suggest that the problem is not due to increased pooling in the venous capacitance vessels, but rather due to decreased blood flow in the skin [16,17].

### Laboratory Abnormalities in POTS

Some authors advocate the use head-up tilt table testing as a standardized method to assess an individual's response to a change in posture [1]. The patient is positioned on a standard tilt table and following baseline measurements of blood pressure and heart rate, the patient is inclined to a 70-degree head-up angle. Blood pressure and heart rate are then measured either continuously or at least every 12 minutes. The orthostatic tachycardia is often measured in a similar fashion to the standing test, with a similar threshold used to diagnose orthostatic tachycardia (an increase of ≥30 bpm) [1]. However, the physiology in response to passive standing on a tilt table (with the legs still) is not the same as“active standing” where the patient must support their own weight and maintain their balance. The latter requires use of the “skeletal muscle pump” and mimics real life, while the tilt table does not. For this reason Streeten et al. use similar criteria for orthostatic tachycardia (>27 bpm), but only with active standing [18]. In a recent study, we compared the orthostatic heart rate response of these 2 methods, and found that the tilt table test was associated with an increased orthostatic tachycardia in both patients with POTS and control subjects [19]. While both tests were sensitive for the diagnosis of POTS with a 30 bpm threshold for orthostatic tachycardia, the stand test had a specificity of 79% compared to only 23% for the tilt table test.

POTS patients should have only sinus tachycardia. An electrocardiogram should be done routinely to rule out the presence of an accessory bypass tract or any abnormalities of cardiac conduction. A Holter monitor might prove useful to exclude a re-entrant dysrhythmia, especially if the patient gives a history of paroxysmal tachycardia with a sudden onset and sudden offset. Other tests such as echocardiograms are only required in individual cases when there is doubt about the structural integrity of the heart.

We often measure plasma norepinephrine levels in both a supine and standing position (at least 15 minutes in each position prior to blood sampling). The supine norepinephrine is often high normal in patients with POTS, while the upright norepinephrine is usually elevated (>600 pg/ml), a reflection of the exaggerated neural sympathetic tone that is present in these patients while upright.

Tests of autonomic nervous system function typically show intact or exaggerated autonomic reflex responses. These patients often have preserved vagal function as reflected by their sinus arrhythmia ratio in response to deep breathing. They often have a vigorous pressor response to the Valsalva maneuver, with an exaggerated blood pressure recovery and overshoot both before and after release [20].

The blood volume is low in many patients with POTS [5]. This can be objectively assessed with nuclear medicine tests to directly measure either the plasma volume or the red cell volume. This knowledge may help to focus the treatment plan.

Some patients with POTS have co-existent complaints of episodic flushing. In about half of these cases there is an associated mast cell activation disorder [20]. This can be diagnosed by collecting urine from individual 2-4 hour voids following a severe flushing spell for determination of methylhistamines.

## Differential Diagnosis

The clinical picture of POTS can be confused with pheochromocytoma because of the paroxysms of hyperadrenergic symptoms. Patients with pheochromocytoma are more likely to have symptoms while lying down than POTS patients, and often have much higher plasma norepinephrine levels. The diagnosis of pheochromocytoma is made by assessment of plasma or urinary metanephrines [21].

There is commonly some confusion between neurally mediated syncope and POTS. There is a clinical overlap between the 2 disorders, such that about 30% of patients with POTS also have neurally mediated syncope. Nonetheless, most patients with POTS do not faint.

Almost all patients with POTS also have associated fatigue. The reasons are not entirely clear. In some patients, but not all, the fatigue improves with pharmacological control of the orthostatic tachycardia. Some patients with POTS have symptomatic overlap with chronic fatigue syndrome.

## Pathophysiology of POTS

**Figure 3:**
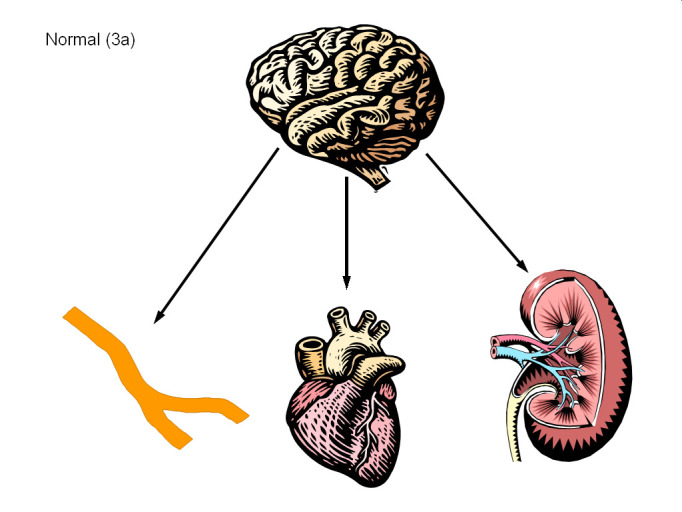

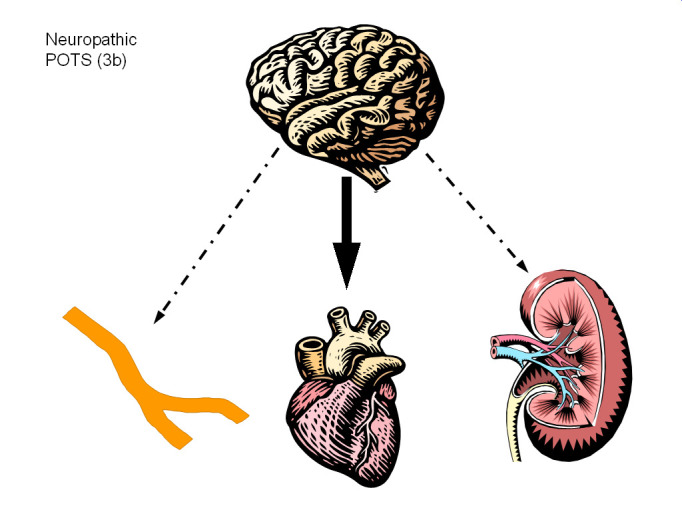

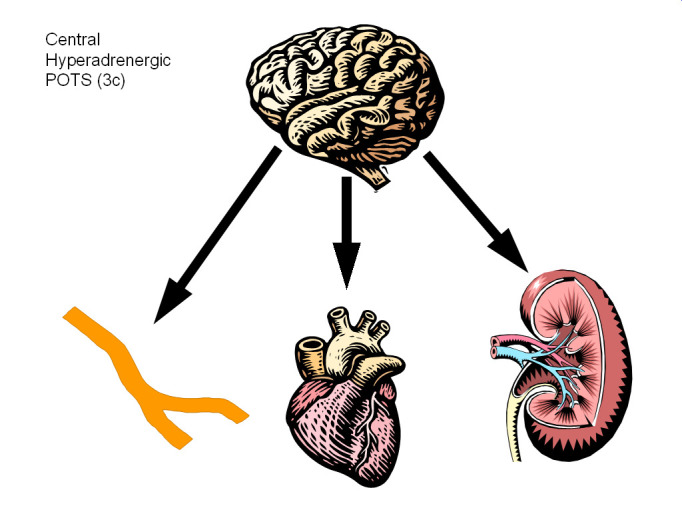
Pathophysiological Schema in POTS There are multiple distinct pathophysiological subtypes within the postural tachycardia syndrome (POTS). The **top panel** (**3a**) shows a basal situation with a normal amount of sympathetic nervous system outflow from the brain that activates receptors in the blood vessels (vascular tone & venous return), heart (heart rate & contractility) and kidney (blood volume regulation through renin). **Panel 3b** shows a schematic of Neuropathic POTS. There is patchy denervation of the sympathetic innervation of the blood vessels in the extremities (especially the legs) and the kidney with subsequent hypovolemia and increased orthostatic venous pooling. This feeds back to the brain to increase sympathetic nervous system outflow in a compensatory effort. This increased sympathoneural flow is sensed most in the heart where there is no denervation. **Panel 3c** shows a schematic of Central Hyperadrenergic POTS. In this case, the underlying problem is excessive sympathetic nervous outflow from the brain that affects the blood vessels, kidneys and the heart. In addition to tachycardia, this form of POTS is often associated with orthostatic hypertension.

Tachycardia and asthenia on standing is a final common pathway of many pathophysiological processes. POTS is therefore best viewed as a syndrome rather than a disease. Many disorders with a common key clinical presentation (the orthostatic tachycardia) have been described. Over the last decade, much has been learned about specific forms or sub-types within POTS, although a simple test to categorize the individual patient remains elusive. We discuss here the common POTS phenotypes including neuropathic POTS and central hyperadrenergic POTS (Figure 3).

### Neuropathic POTS

Considering that POTS patients have high plasma NE levels, it would seem paradoxical that a neuropathy is proposed as an underlying process. Yet some of them have a form of dysautonomia, with preferential denervation of sympathetic nerves innvervating the lower limbs [22-24]. There have been several findings consistent with this hypothesis. The results of sudomotor axon reflex testing [22] and galvanic skin stimulation [23] support this as well as skin biopsy results [25]. Further, these patients have been found to be hypersensitive to infusions of norepinephrine and phenylephrine into veins of the foot, despite high circulating plasma norepinephrine concentrations [24]. This suggests that there is a denervation hypersensitivity of the leg veins. Using a segmental norepinephrine spillover approach, Jacob et al. [26] demonstrated that patients with POTS had normal sympathetic neuronal norepinephrine release in their arms, but less norepinephrine release (and thus less sympathetic activation) in their lower body.

### Hypovolemia & Blood Volume Regulation

**Figure 4:**
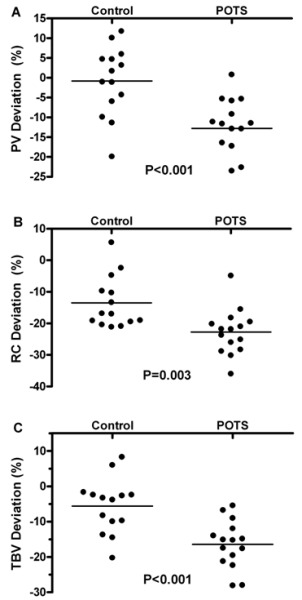
Blood Volume Deviation in POTS The 3 panels show the blood volumes of control subjects and patients with POTS compared to hat expected based on their individual height, weight and gender. Data are shown for plasma volume (**PV; Panel A**), red cell volume (**RC; Panel B**) and total blood volume (**TBV; Panel C**). The plasma volume and total blood volume of the control subjects was similar to their expected values. The patients with POT had a deficit of their plasma volume (**Panel A**), red cell volume (**Panel B**) and total blood volume (**Panel C**) compared to the control group. Figures adapted with data from Raj SR, Biaggioni I, Yamhure PC, Black BK, Paranjape SY, Byrne D, Robertson D. The Renin-Aldosterone Paradox and Perturbed Blood Volume Regulation Underlying the Postural Tachycardia Syndrome. Circulation 2005; 111:1574-1582.

Many patients with POTS have low plasma volumes [27,28], but not all. To determine if hypovolemia existed in an unselected group of POTS patients, we studied 15 patients with POTS (not selected for blood volume) and 14 control subjects [5]. Plasma volume was measured using ^131^I labeled human serum albumin using a dye dilution technique, and compared to the predicted blood volume for each individual, based upon their height, weight, and gender. As can be seen in Figure 4, the control subjects did not have a significant plasma volume deficit (0.8±2.5%). In contrast, the patients with POTS had a plasma volume deficit of 12.8±2.0% (P<0.001).

The renin-angiotensin-aldosterone system plays a key role in the neurohormonal regulation of plasma volume in humans. Plasma renin activity and angiotensin II would be expected to increase in response to hypovolemia in order to promote blood volume expansion. Angiotensin II promotes sodium and water retention directly by stimulating sodium resorption in the proximal tubules, and indirectly by stimulating aldosterone secretion.

Patients with orthostatic tachycardia who were also hypovolemic have low levels of standing plasma renin activity and aldosterone compared to normovolemic patients [21,22]. This is true in both supine (190±140 pM vs. 380±230 pM; P=0.017) and upright posture (480±290 pM vs. 810±370 pM; P=0.019). One would have expected a compensatory increase in both plasma renin activity and aldosterone given the hypovolemia in these patients. This low level of plasma renin activity and aldosterone is a paradox that remains unexplained. These data suggest that abnormalities in the renin-angiotensin-aldosterone axis might have a role in the pathophysiology of POTS by contributing to hypovolemia and impaired sodium retention. Such hypovolemia could be accounted for by a neuropathic process involving the kidney. A significant modulator of renin release is the sympathetic nervous system. Thus perturbations in the renin-aldosterone system might result from partial sympathetic denervation involving the kidney.

### Central Hyperadrenergic POTS

As a part of the definition, POTS is associated with a hyperadrenergic state (Table 1). In many such cases, the hyperadrenergic state is secondary to a partial dysautonomia or hypovolemia. There are some cases, however, in which the primary underlying problem seems to be excessive sympathetic discharge. These patients often have extremely high levels of upright norepinephrine. While we require the upright norepinephrine level to be >600 pg/ml for the diagnosis of POTS, the hyperadrenergic subgroup often has upright norepinephrine level >1000 pg/ml and it is occasionally >2000 pg/ml. These patients sometimes have large increases in blood pressure on standing, indicating that baroreflex buffering is somehow impaired.

Central hyperadrenergic POTS in its most florid form is much less common than neuropathic POTS, comprising only ~10% of patients. Thus therapy in these cases usually targets a decrease in sympathetic tone both centrally and peripherally.

Central sympatholytics such as methyldopa or clonidine can be used. Peripheral beta-adrenergic blockade may be better tolerated by these patients than by those with neuropathic POTS.

### Norepinephrine Transporter Deficiency

A specific genetic abnormality has been identified in a kindred with hyperadrenergic POTS [30]. These individuals have a single point mutation in the norepinephrine transporter (NET). The resultant inability to adequately clear norepinephrine produces a state of excessive sympathetic activation in response to a variety of sympathetic stimuli. While rare, this mutation has taught us much about the importance of a functional NET.

Although functional NET mutations might be infrequent, pharmacological NET inhibition is very common. Many antidepressant and attention deficit medications work at least in part through inhibition of NET. This includes traditional drugs such as tricyclic antidepressants, and newer medications which are pure NET inhibitors (e.g. atomoxetine or reboxetine). Both we [31] and others [32] have found that pharmacological NET inhibition can recreate an orthostatic tachycardia phenotype in susceptible healthy volunteer subjects. Yohimbine, a central alpha-2 antagonist that will also increase synaptic norepinephrine, can also cause orthostatic tachycardia [33].

### Mast Cell Activation

Some patients with POTS have co-existent mast cell activation. These patients have episodic flushing and abnormal increases in urine methylhistamine (the primary urinary metabolite of histamine) [20]. Methylhistamine should ideally be measured in 2 hour aliquots at the time of a flushing episode and not just in a random 24 hour period. Other associated symptoms include shortness of breath, headache, lightheadedness, excessive diuresis, and gastrointestinal symptoms such as diarrhea, nausea, and vomiting. Flushing can be triggered by long-term standing, exercise, premenstrual cycle, meals, and sexual intercourse. These patients often have a hyperadrenergic response to posture, with both orthostatic tachycardia and hypertension. They demonstrate a vigorous sympathetic vasopressor response during the Valsalva maneuver with a blood pressure overshoot in late phase II and an exaggerated phase IV blood pressure overshoot. It is not clear if mast cell activation, releasing vasoactive mediators, represents the primary event in these patients or if sympathetic activation, through release of norepinephrine, neuropeptide Y and ATP, is the cause of mast cell activation [34].

In these patients, beta-adrenergic antagonists can actually trigger an episode and worsen symptoms. Centrally acting agents to decrease the sympathetic nervous system discharge (e.g. methyldopa or clonidine) may prove effective. Alternatively, treatment could target mast cell mediators with a combination of antihistamines (H1- and H2-antagonists) and with the cautious use of non-steroidal agents (high dose aspirin) in refractory cases.

## Non-Pharmacological Treatment of POTS

No therapy is successful for all patients with POTS. Initial efforts should focus on identifying and treating any reversible causes. Potentially contributory medications (especially vasodilators, diuretics, and drugs that inhibit NET) should be withdrawn. If a patient has been through prolonged bedrest, their symptoms will gradually improve as they recondition themselves to upright posture. Treatment should be optimized for any chronic disease that is present. If there is clear evidence of a re-entrant supraventricular arrhythmia, then this should be treated, including with radiofrequency ablation as appropriate. However, radiofrequency sinus node modification for the sinus tachycardia of POTS is not recommended. This often makes the patient’s symptoms worse (and occasionally the patient becomes pacemaker dependent). Specific therapies are summarized in Table 2.

It is important to educate the patient about the nature of the disorder. The patient should avoid aggravating factors such as dehydration, and extreme heat. In order to ensure adequate hydration, we ask our patients to consume 8-10 cups of water daily and to rapidly drink 16 fl oz of water to lower their heart rates [35]. In addition, they are asked to aggressively increase their sodium intake up to 200 mEq/day. This is often hard to achieve without NaCl tablets 1 gm/tablet TID with meals. Elastic support hose can help to minimize the degree of peripheral venous pooling and enhance venous return. We recommend 30-40 mmHg of counter-pressure and they should come up to the waist. If the stockings are only knee-high, a line of edema can form just above the stockings. Their use can be limited by their tolerability as the stockings can be hot, itchy and uncomfortable. Exercise (both aerobic and resistance training) is also encouraged and has been shown to be beneficial [36]. In addition to reversing any “deconditioning”, this intervention can also increase blood volume. Vigorous exercise may acutely worsen symptoms and may even result in prolonged fatigue. It is important that patients start slowly and remain within range of their “target heart rate” in the early stages to avoid symptoms that might discourage further exercise.

Acute blood volume expansion is effective at controlling the heart rate and acutely improving symptoms. Jacob et al. [37] found that 1 liter of physiological saline infused intravenously over 1 hour decreased the orthostatic tachycardia from 33±5 bpm before the infusion to 15±3 bpm immediately following the infusion. The physiological saline was more effective at heart rate control than were treatments with either an alpha-1 agonist or an alpha-2 agonist. This treatment is not practical on a day to day basis as a medical setting is required to insert the intravenous catheter and infuse the saline. Recently, there have been reports of patients having regular saline infusions, typically 1 liter of normal saline every other day or every day. Many report an improvement in symptoms. However, there are not yet objective data to substantiate such benefit. Further, there is a risk of vascular access complications or infection. At this time, such therapy for patients with POTS should be considered cautiously.

## Pharmacological Treatment of POTS

No medicines are approved by the United States Food and Drug Administration for the treatment of POTS. Thus all agents are used for this disorder are “off label”. Furthermore, there are no pharmacological agents that have been tested in a long-term properly powered randomized clinical trial.

In patients in whom the presence of hypovolemia is either known or strongly suspected, fludrocortisone (an aldosterone analogue) is often used. Through enhanced sodium retention, it should expand the plasma volume, although there is a paucity of data regarding the exact mechanisms of action. Although fairly well tolerated, side effects can include hypokalemia, hypomagnesemia, worsening headaches, acne, and fluid retention with edema. Another volume expanding agent that may be helpful for short-term use is oral vasopressin (DDAVP). This agent causes the kidney to retain free water, but not sodium. Potential side effects include hyponatremia, edema and headache. Erythropoietin has occasionally proven useful in patients with POTS who are refractory to other forms of therapy. While the primary mode of action is likely an increase in intravascular volume via its increase in red cell mass, erythropoietin also appears to have a direct vasoconstrictive effect, possibly through enhanced red cell mediated nitric oxide scavenging [38]. Treatment with erythropoietin has many drawbacks including the significant expense and the need for subcutaneous administration.

Central sympatholytic medications are often useful and well tolerated in patients with the central hyperadrenergic form of POTS, but may not be as well tolerated in neuropathic POTS. Clonidine is an alpha 2 agonist that acts centrally to decrease sympathetic nervous system tone. Clonidine, at doses of 0.05 mg to 0.2 mg PO BID, can stabilize heart rate and blood pressure in patients with a large amount of postganglionic sympathetic involvement. Unfortunately, it can also cause drowsiness, fatigue and worsen the mental clouding of some patients. Methyldopa, a false neurotransmitter, is sometimes more successful in controlling symptoms in these patients at doses of 125 mg to 250 mg PO TID [39].

When used in low doses, beta-adrenergic antagonists can be useful. We typical use propranolol 10-20 mg PO BID-QID. While this dose range is small, such doses can often have a significant impact on heart rate control, and higher doses are often not tolerated due to hypotension and fatigue.

Since a failure of vascular resistance may be an integral part of neuropathic POTS, vasoconstrictors such as midodrine (an alpha-1 agonist) can be employed [40]. Some patients cannot tolerate this agent due to the unpleasant sensation of scalp tingling or goosebumps. Midodrine can also cause hypertension.

We recently reported that an unselected group of patients seen in our inpatient research unit were given a trial of the acetylcholinesterase inhibitor pyridostigmine. By increasing the levels of synaptic acetylcholine at both the autonomic ganglia and the peripheral muscarinic parasympathetic receptors, pyridostigmine significantly restrained the heart rate in response to standing in our patients with POTS. We prescribe pyridostigmine 30mg to 60 mg PO TID alone or in combination with low dose propranolol. Pyridostigmine can enhance bowel motility, so it is not always well tolerated in patients with diarrhea-predominant irritable bowel syndrome symptoms.

While most of the treatments discussed above have focused on the control of heart rate, many patients are also greatly troubled by mental clouding. Modafinil, a stimulant whose mechanism is not yet clear, has been used in some patients with resulting improvement in alertness. However, caution is advised as it may aggravate the orthostatic tachycardia [41].

## Conclusions

POTS is a disorder of the autonomic nervous system in which many symptoms can be treated. The cardinal manifestation is symptomatic orthostatic tachycardia. The disorder can produce substantial disability among otherwise healthy people. Patients with POTS demonstrate a heart rate increase of ≥30 bpm with prolonged standing (5-30 minutes), often have high levels of upright plasma norepinephrine, and many patients have a low blood volume. Therapies aimed at correcting the hypovolemia and the autonomic imbalance may help relieve the severity of the symptoms. Continued research is vital to better understand this disorder and to differentiate its various subtypes.

## Figures and Tables

**Table 1:**
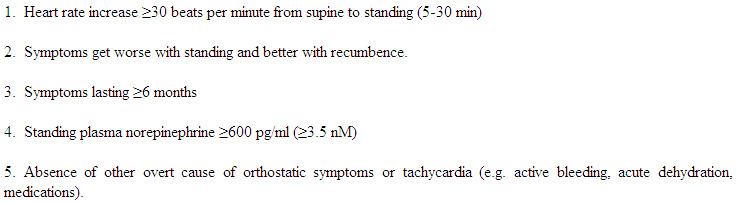
Criteria for the Postural Tachycardia Syndrome

**Table 2:**
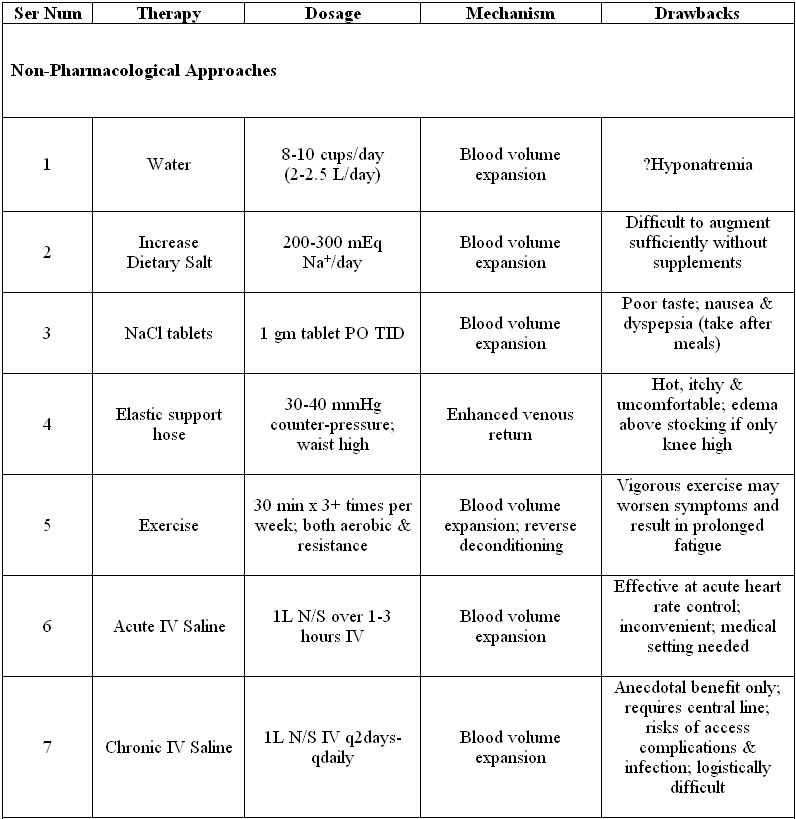

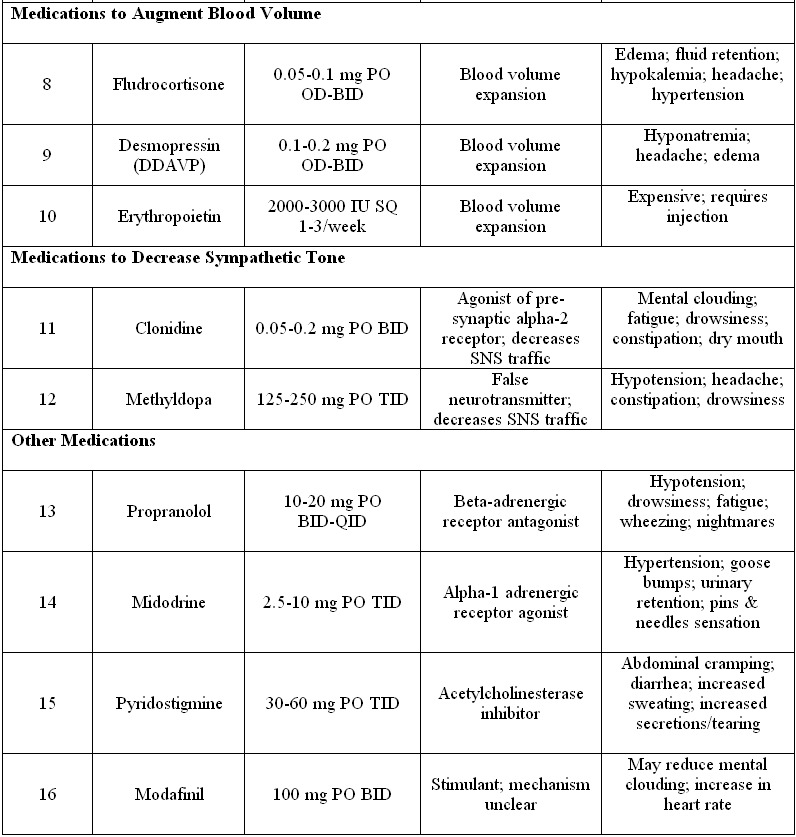
Treatments for the Postural Tachycardia Syndrome

NaCl - Table salt; PO - by mouth; OD - once daily; BID - twice daily; TID - three times daily; QID - four times daily; IV - intravenous
